# Ventricular Tachycardia Originating from Moderator Band: New Perspective on Catheter Ablation

**DOI:** 10.1155/2017/3414360

**Published:** 2017-01-18

**Authors:** Jin-yi Li, Jing-bo Jiang, Yan He, Jian-chun Luo, Guo-qiang Zhong

**Affiliations:** ^1^Department of Cardiology, The First Affiliated Hospital of Guangxi Medical University, Nanning 530021, China; ^2^Department of Cardiology, The First People's Hospital of Guilin, Guilin 541002, China; ^3^Department of Cardiology, The 303 Hospital of the Chinese People's Liberation Army, Nanning 530021, China

## Abstract

A 59-year-old woman was referred to the institution with burdens of idiopathic ventricular tachycardia (IVT). Electroanatomic mapping revealed a complex fractionated, high frequency potential with long duration preceding the QRS onset of the IVT. The real end point of ablation was the disappearance of the conduction block of Purkinje potential during the sinus rhythm besides the disappearance of the inducible tachycardia. Location of distal catheter was at the moderator band (MB) by transthoracic echocardiography (TTE). Only irrigated radiofrequency current was delivered at both insertions of the MB which can completely eliminate the IVT.

## 1. Introduction

Idiopathic ventricular tachycardia (IVT) usually emerges in the patients without structural heart disease or electrical abnormalities. Triggers for premature ventricular complexes (PVCs) and IVT are mostly located at the right and left ventricular outflow tract, the His-Purkinje fibers system, and atrioventricular valves. Some regions such as papillary muscles and anterior wall of the right ventricle also account for the triggers of PVCs and IVT. Although previous studies had proved the efficacy of radiofrequency ablation for most IVT, a small series of IVT arising from uncommon regions are difficult to be ablated and also are underestimated. Anter et al. [1] reported the first case of VF originating from the moderator band (MB), a structure that never was thought to have a correlation with arrhythmia before. Sadek et al. [2] then summarized a small series of IVT arising from the MB. Both of these studies described different targets for ablation that needs further discussion. Here we report an IVT originating from the MB undergoing catheter ablation with a precise target.

## 2. Case Report

A 59-year-old, previously healthy woman was referred to the institution for repeated palpitations for six years. Within the last twelve days, the symptoms became severe, even with once presentation of syncope at home. A Holter ECG from the local hospital showed 29854 events of ventricular arrhythmia (17% of the total heart beats), including 3444 episodes of nonsustained ventricular tachycardia, 754 ventricular couplets, and 1654 single premature ventricular ectopics. Initial therapy with oral antiarrhythmia drugs (amiodarone and propafenone) was unsuccessful.

**Figure 1:**
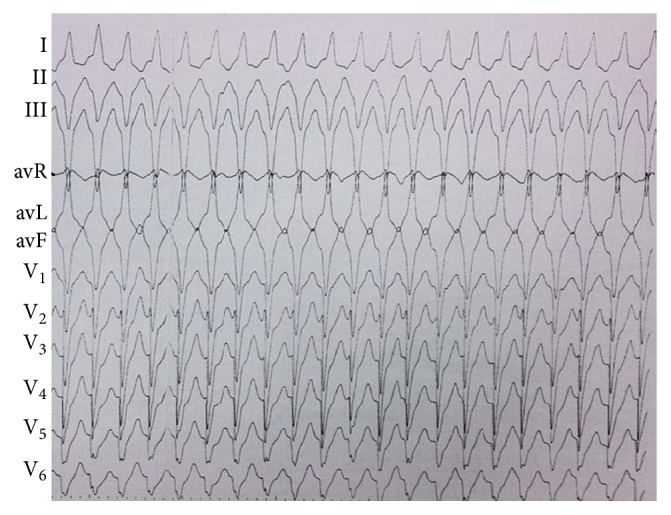
Twelve-lead ECG of IVT.

Initial workup included baseline ECG, normal blood test, chest X-ray examination, echocardiogram, and coronary angiography. The frequent unifocal initiating IVT had a unique morphology characterized by a left bundle branch block (LBBB) pattern with very late precordial transition and a left superior axis (Figure 1).

**Figure 2:**
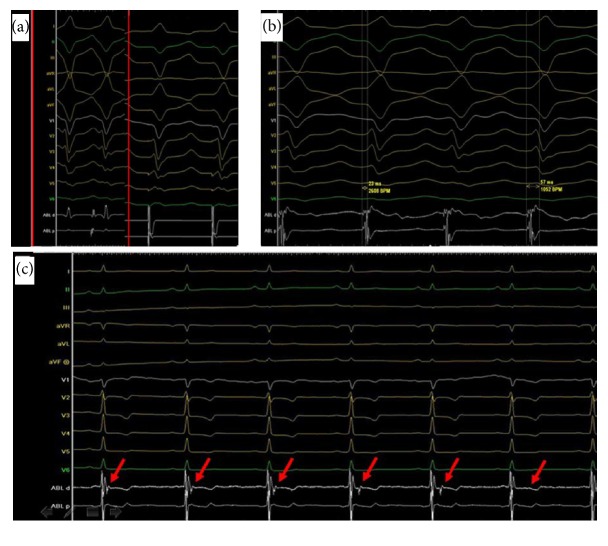
(a) Pacing mapping showed that the morphology of pacing QRS complex is identical to the QRS complex of clinical IVT (inside the red box). (b) A complex fractionated, high frequency potential with long duration of 57 milliseconds, preceding the QRS onset of the IVT by 23 milliseconds. (c) A conduction block of Purkinje potential (red arrow) was observed during the sinus rhythm after successful ablation during the first procedure.

**Figure 3:**
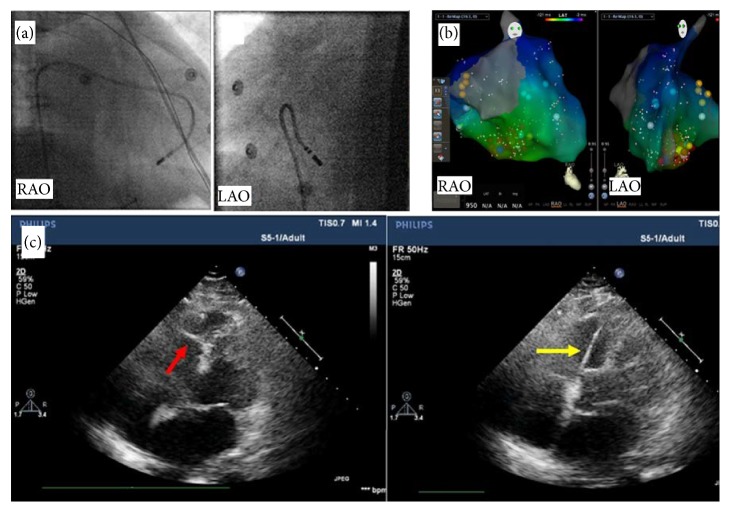
(a) Ablation catheter at the earliest activation site on the MB using fluoroscopy. (b) Electroanatomic mapping showed that the ablation zone (red points) was placed at the site of earliest activation at the middle RV of lower septum. (c) Ablation at the earliest activation site on the MB (red arrow) guiding by TTE; the ablation catheter (yellow arrow) was placed at the septum insertion of MB. RAO: right anterior oblique; LAO: left anterior oblique.

After providing informed consent, the patient underwent electrophysiologic study and ablation while antiarrhythmic medications had been held for 2 half-lives. Endocardial mapping of the right ventricle (RV) was performed by using a 3.5 mm open irrigated-tip catheter (Navistar Thermocool, Biosense Webster, Diamond Bar, CA, USA) and CARTO mapping system (Carto, Biosense Webster). Transthoracic echocardiography (TTE) was applied to define the anatomy, facilitate mapping, and assess contact during ablation. A CARTO electroanatomic mapping system (Biosense Webster) was used. Spontaneous PVCs/IVT were identified and mapped when present by activation mapping. A complex fractionated, high frequency potential with long duration of 57 milliseconds, preceding the QRS onset of the arrhythmogenic VT by 23 milliseconds, was mapped at the site of the earliest myocardial activation (Figure 2(b)). This potential was systematically mapped from the middle RV at the lower septum, along the papillary muscle to the insertion at the RV apex and the extension to the interventricular septum (Figures 3(a) and 3(b)) via the MB. Pacing mapping showed that the morphology of pacing QRS complex is identical to the QRS complex of clinical VT (Figure 2(a)). The anatomical site of the earliest activation and the location of distal catheter were at the MB as visualized by TTE (Figure 3(c)).

Radiofrequency ablation (RFCA) current was delivered at 30 W of power-controlled mode with a temperature of 43°C and a normal saline velocity of 17 milliliters per hour. RFCA was delivered at the free-wall insertion along the MB which can successfully terminate the VT, with a conduction block of Purkinje potential observed during the sinus rhythm (Figure 2(c)). No ventricular arrhythmia can be induced during program stimulation and isoproterenol infusion (10 *μ*g/min). The patient had no further episodes of IVT but few asymptomatic PVCs over the ensuing 120 days until her discharge home without antiarrhythmic drugs. Three months later, the patient was hospitalized again because of recurrent symptomatic tachycardia. Surface ECG showed the morphology of the recurrent IVT had no change with the original one. The patient underwent a second ablation, which employed a similar mapping system and the ablation energy to the first procedure. TTE was also applied for the procedure to visualize and guide the catheter for the contact of MB. Different from the first procedure, RFCA was delivered at the septum insertion along the MB with an end point of the disappearance of the conduction block of Purkinje potential during the sinus rhythm besides the disappearance of the inducible tachycardia. The patient had no further episodes of IVT over the ensuing 15 months until her discharge home without antiarrhythmic drugs.

## 3. Discussion

MB is part of the septomarginal trabeculation, supporting the anterior papillary muscle of the tricuspid valve, and connects the anterior papillary muscle to the free wall of the ventricle. It was thought to be a structure protecting against overdistention of the RV, hence the name “moderator” [3]. The role of the MB as part of the conduction system of the heart involves the right atrioventricular bundle, as conduction tissue fibers move toward the apex of the ventricle before entering the anterior papillary muscle [4]. The morphology and the topology of the MB had significant diversity [5]. The possibility of relationship between the abnormity of the morphology and the topology of MB and ventricular arrhythmia need further research.

For the histological examinations of MB, clumps of conductive cells and large amounts of muscle fibers were identified. Conductive tissue was represented by clumps of Purkinje cells, surrounded by myocardial fibers [6]. The rich autonomic innervations of the MB may contribute to its mechanism of arrhythmogenicity. In our report, a conduction block of Purkinje potential was observed after temporary terminating the IVT.

Information about the prevalence, ECG features, and results of ablation of MB PVCs is limited. An article from Anter et al. reported a case of a 59-year-old man with idiopathic ventricular fibrillation (VF) storm. As visualized by intracardiac echocardiography (ICE), radiofrequency ablation at the MB, recording the earliest Purkinje potential separated from the local electrogram, was successful [1]. The author believed that the trigger was originated from the RV Purkinje network because ablation at the earliest Purkinje potential was successful. We partly agree with this view. We also recorded a Purkinje potential during ablation, of which conduction block was observed after temporary terminating the IVT. But the ablation of Purkinje potential target only was insufficient for terminating VT. The complex fractionated, high frequency potentials with long duration preceding the QRS onset of the arrhythmogenic VT were exactly our ablation targets, which suggest that the mechanism of microreentry among the Purkinje system and the connected myocardia is reasonable. Sadek et al. [2] summarized an MB source of PVCs in 10 patients presenting with VAs that were mapped to the MB, 7 of whom presented with PVC-induced VF. VAs originating from the MB have LBBB morphology with a left superior frontal plane axis, a sharp downstroke of the QRS in the precordial leads, and a relatively narrow QRS width. Not only does MB ventricular arrhythmia have a late precordial transition pattern, typically after V4, but also the transition is always later than that of the sinus QRS. The QRS morphology occasionally changed during ablation, suggesting a change in the exit site. The site of successful ablation location along the MB was variable, including the septum insertion, the body of the MB, and the free-wall insertion. 60% of patients with MB originating PVCs required a second ablation procedure days to months after the initial procedure. According to our experiences of two procedures of ablation, both of the septum insertion and the free-wall insertion of MB should be ablated together in order to completely eliminate the IVT. The real end point of ablation was the disappearance of the conduction block of Purkinje potential during the sinus rhythm besides the disappearance of the inducible tachycardia. A reasonable explanation for these perspectives is that MB originating IVT has two exits including the septum insertion and the free-wall insertion of MB. Ablation of only one insertion would lead to a conduction block of Purkinje potential and a temporary termination of IVT. Ablation of both insertions of MB is necessary to completely eliminate the IVT with a disappearance of the conduction block of Purkinje potential.

The contact and the stability of catheter are vital to the successful ablation. ICE is the most effective way of visualization for the MB during ablation, which is widely used for ablation on the papillary muscles. In our report, we successfully used the TTE to visualize and guide the catheter for the contact and the stability during ablation, which is of high cost efficiency for the patient. We found the MB and the catheter can be monitored dynamically during ablation, which is of importance for the ablation of MB not only on the septum insertion but also on the free-wall insertion. Cryoablation and contact force technology may be quite useful for achieving greater success when ablating on the MB [2].

Medical therapy for MB originating IVT is limited. Lidocaine [1], sedation [1], propafenone, and amiodarone are unsuccessful for MB originating IVT before catheter ablation. Quinidine [2] and propafenone seem to be effective on MB originating PVCs after catheter ablation without VT and VF. Because VF is commonly detected or induced as previously reported, an implantable cardioverter-defibrillator (ICD) therapy for secondary prevention or even primary prevention before and after unsuccessful catheter ablation is lacking feasibility report. A large sample research is needed in the future. The prognosis of MB originating IVT without any therapy is uncertain. But we do suggest that the interventional therapy should be positively applied to prevent the malignant arrhythmia including cardiac arrest. And, although catheter ablation seems to be effective in eliminating the IVT, ICD first or catheter ablation first is still a question.
